# The Reliability and Validity of the Malay Version of Polycystic Ovarian Syndrome Health-Related Quality of Life Questionnaire

**DOI:** 10.3389/fendo.2022.848860

**Published:** 2022-05-26

**Authors:** Lim Leek Mei, Muhammad Azrai Abu, Kah Teik Chew, Aniza Ismail, Ani Amelia Zainuddin, Abdul Ghani Nur Azurah

**Affiliations:** ^1^ Department of Obstetrics and Gynaecology, Hospital Sultan Ismail, Johor Bahru, Malaysia; ^2^ Advanced Reproductive Centre, Universiti Kebangsaan Malaysia (UKM) Medical Centre, Kuala Lumpur, Malaysia; ^3^ Department of Obstetrics and Gynaecology, Universiti Kebangsaan Malaysia (UKM) Medical Centre, Kuala Lumpur, Malaysia; ^4^ Department of Public Health, Universiti Kebangsaan Malaysia (UKM) Medical Centre, Kuala Lumpur, Malaysia

**Keywords:** polycystic ovary syndrome, Polycystic Ovary Syndrome Questionnaire, Malay version PCOSQ, health-related quality of life, reliability, validity

## Abstract

The Polycystic Ovary Syndrome Questionnaire is a reliable instrument for measuring health-related quality of life. This study aimed to develop a Malay version of the Polycystic Ovary Syndrome Questionnaire and to evaluate the health-related impact of Malaysian women with polycystic ovary syndrome. The participants were women who were diagnosed with polycystic ovary syndrome using Rotterdam criteria in a gynecology clinic. Reliability was determined by internal consistency using Cronbach’s coefficient alpha and test–retest reliability using an intra-class correlation coefficient. Validity was assessed through convergent and discriminant validity. Examining the correlation between similar content of the Malay version of the Polycystic Ovary Syndrome Questionnaire and the SF-36 assessed the convergent validity. The discriminant validity was assessed using the known group comparison. Cronbach’s alpha coefficient was over 0.70 for the total scale and over 0.60 for each subscale. Known group comparison supported the discriminant validity. The Malay version of the Polycystic Ovary Syndrome Questionnaire differentiated between the subgroups of women who differed in polycystic ovary syndrome-specific symptoms. Convergent validity was consistent with the good positive correlation between related subscales of the two instruments. Polycystic ovary syndrome women in Malaysia scored the lowest for the weight (3.74) and infertility (3.41) domains, thereby indicating worse health status in these domains. Body hair (5.42) was the least troublesome for the local population. The Malay version of the Polycystic Ovary Syndrome Questionnaire is a reliable and valid tool for assessing the health-related quality of life among women in the local population. It can be used to objectively assess the quality of life among Malaysian women with polycystic ovary syndrome and evaluate their responsiveness to treatment modalities.

## Introduction

Polycystic ovary syndrome (PCOS) is the most common endocrine condition that affects women, with an estimated prevalence of 10–15% (1–3). Due to its heterogeneity, PCOS may manifest as a broad-spectrum feature with varying severity. It is commonly associated with menstrual cycle disturbances, hirsutism, acne, alopecia, obesity, and fertility issues (4–7). The introduction of Rotterdam criteria, which consist of amenorrhea or oligomenorrhea, hyperandrogenism features, and sonographic evidence of polycystic ovaries, allowed the exclusion of other possible causes of menstrual disturbance and androgen excess. Women with PCOS may show profound general health implications, mainly those related to psychological wellbeing, and these are usually neglected by clinicians (8–10).

The symptoms that are typically associated with PCOS have led to a significant reduction in health-related quality of life (HRQoL) (11–14). The affected aspects of HRQoL differ individually, as each woman may present with different concerns related to PCOS. Adult women are mainly concerned about subfertility issues and hirsutism, whereas adolescents are more likely to worry about acne and weight gain (15–18). The development of a disease-specific questionnaire, such as the Polycystic Ovarian Syndrome Questionnaire (PCOSQ) by Cronin et al., has led to the proper assessment of specific issues affecting women with PCOS. The lowest score from each subscale reflected a poor quality of life (19).

The impact of PCOS on the quality of life varied among women from different populations and backgrounds worldwide despite its specificity. The PCOSQ was found to be reliable among Iranian and Arab populations, but it was lacking in the menstrual problem domain. In the UK and Chinese populations, all domains were valid, reliable, and culturally acceptable; however, it can be improved by adding the acne dimension (20–24). Therefore, a translation of the PCOSCQ is compulsory for adjusting the cultural and language differences in various countries.

The validation of the Malay version has not been conducted; hence, no data are available on the health-related QoL of the Malaysian population. The aim of this study was to demonstrate the reliability and validity of the Malay version of PCOSQ. Furthermore, this questionnaire would allow comparisons of results between different regions, thereby leading to the improvement of the HRQoL of women with PCOS.

## Materials and Methods

### Study Design

A prospective cohort study was conducted at the University Kebangsaan Malaysia Medical Centre (UKMMC) from June 2018 to June 2019. All women aged 18 to 45 years old who were diagnosed with PCOS following the Rotterdam criteria were recruited. Women with psychiatric illnesses were excluded.

### Study Tools

The original PCOSQs were published in the *Journal of Clinical Endocrinology and Metabolism* by The Endocrine Society in 1998 (19). The permission to translate the original questionnaires was obtained through the Copyright Clearance Center. Two Malay translators from the Malaysian National Institute of Translations who were competent in English translated the PCOSQ into the Malay language independently. Following the forward translation, a discussion was held between the translators to develop a single Malay version. Afterward, two other translators who were both blinded from the original version performed a backward translation from Malay to English. The original English version and the translated Malay version were assessed, and no major discrepancy was identified. A pilot study was conducted on 20 women to assess their response and understanding of each question and to determine the duration of completing the questionnaire.

#### SF-36v2

The SF-36v2 is the latest version of SF36. It is a generic indicator of health status and has been applied to the assessment of outcomes of various health conditions in the general population. The instrument has been tested for validity and reliability. The SF-36v2 measured and included eight subscales as follows: physical functioning, role limitations due to physical problems, bodily pain, general health perception, social functioning, role limitations due to emotional problems, vitality, and mental health. The score for each subscale ranged from 0 to 100. A higher score indicated a better condition. A Malay version is available; it is deemed reliable and valid for use (**Appendix 1**).

#### Mal-PCOSQ

The original PCOS health-related quality of life questionnaire is in English; it was published by Cronin et al. (19) in 1998 in the USA. It includes 26 items in five domains, and it takes 10–15 min to complete. The domains are divided into emotions (8 items), body hair (5 items), weight (5 items), infertility (4 items), and menstrual problems (4 items). Each question is associated with a 7-point scale, in which 7 represents optimal function and 1 represents the poorest function. The analysis from this study found that this PCOSQ is easy to understand and use. It is recommended that investigators weigh the items equally and present the results as the mean score per item for each of the domains (**Appendix 2**).

### Data Collection

Patients were given an information sheet regarding the study, and they provided their consent. The patients’ demographic data and the details of their menstrual and infertility history were obtained. Body mass index was calculated from the measurement of weight and height. Hirsutism is assessed clinically by using a pictorial scoring system of nine areas of the body following the modified Ferriman–Gallwey score. A score of 0 indicated no terminal hair, whereas a score of 4 indicated severe hirsutism. A total score of 7 or more signified hirsutism. Eligible women were asked to complete the sets of questionnaires that included SF-36v2 and Mal-PCOSQ while waiting to see the doctor. The collected questionnaires were checked for completeness.

### Statistical Analysis

The reliability of the Mal-PCOSQ was assessed by internal consistency using Cronbach’s alpha. A value of >0.70 was considered acceptable. Discriminant validity was assessed using correlation analysis between Mal-PCSOQ and known group comparisons in body hair using the Ferriman–Gallwey score, menstrual irregularities, subfertility, and body mass index. To test for convergent validity, an estimation was calculated between Mal-PCOSQ and SF-36v2 by using Pearson’s correlation, as both are reliable tools for measuring the quality of life. It was hypothesized that a strong correlation exists between the emotional disturbance domains of the Mal-PCOSQ and the role of emotion and mental health domains of SF-36v2. A correlation value of >0.40 was considered good. For predictors of a low score, a logistic regression analysis will be used for categorical independent variables, whereas continuous data will be analyzed using linear regression. For descriptive analyses, the mean, standard deviation, minimum and maximum scores, and skewness were calculated. All statistical analyses of the study were conducted by SPSS 20.0 Windows (SPSS, Inc., USA), and the significance level was set at 0.05.

### Ethical Considerations

Ethical approval was obtained from the Medical Research and Ethics Committee, Universiti Kebangsaan Malaysia Medical Center (FF-2018-020).

## Results

### Demographic Data

A total of 138 participants were recruited, and all completed the questionnaires with no missing data. The mean age of patients was 31.5 ± 4.6 years old. Most participants were Malay, followed by Chinese and Indian participants. Many were obese, with a mean body mass index of 30.6 ± 6.56 kg/m^2^ and a mean waist circumference of 33.28 ± 4.44 cm. About 59.7% of the participants had irregular cycles, and 53.6% had a Ferriman–Gallway score >8; 79% had issues with subfertility (**Table 1**).

**Table 1:** Socio-demographic characteristics.

Socio-demographic characteristics		Mean (SD)	Frequency (%), *N* = 138
Age		31.50 (4.660)	
Ethnicity			
	Malay		84.8
	Chinese		8.0
	Indian		5.8
	Punjabi		1.4
BMI (kg/m^2^)		30.60 (6.56)	
	Underweight (<18.9)		1.4
	Normal (18.9–22.9)		8.7
	Overweight (23–24.9)		8.7
	Obese (>25)		81.2
Waist circumference (cm)		33.28 (4.44)	
Menstrual cycle			
	Regular		40.6
	Irregular		59.4
Ferriman–Gallway score			
	<8		46.4
	>8		53.6
Infertility problem			
	No		21.0
	Yes		79.0

### Reliability

The internal reliability test is shown in **Table 2**. The Cronbach’s alpha was above 0.70; all domains had values above 0.80, except for the menstrual problem domain, which had a Cronbach’s alpha of 0.678. This implied satisfactory reproducibility.

**Table 2:** Reliability test of Mal-PCOSQ.

Domains of MPCOSQ	Cronbach’s alpha
Emotion	0.876
Body hair	0.927
Weight	0.916
Infertility	0.864
Menstrual problem	0.678

### Type of HRQoL Impairment

The distribution of scores for Mal-PCOSQ and SF-36 are listed in **Table 3**. The lowest scores for Mal-PCOSQ were reported in the weight (3.74) and infertility (3.41) domains, thereby indicating worse health in these domains. Body hair (5.42) was the least concerning issue. For SF-36v2, the mental component summary (44.83) and physical component summary (50.15) were both rated low. The highest mean score was seen in physical function (78.51), while vitality (55.75) and general health (57.42) had the lowest scores. The percentages of women who showed the lowest scores (i.e., floor effect) and the highest scores (i.e., ceiling effect) were low.

**Table 3:** Mal-PCOSQ and SF-36 scores and floor and ceiling effects.

	Domains	Mean (SD)	95% CI	Minimum (% floor)	Maximum (% ceiling)
Mal-PCOSQ					
	Emotions	4.29 (1.29)	4.07–4.50	1.4	1.4
	Body hair	5.42 (1.46)	5.18–5.67	0.7	1.8
	Weight	3.74 (1.52)	3.49–4.00	2.9	4.3
	Infertility	3.41 (1.47)	3.16–3.63	4.3	2.2
	Menstrual	4.57 (1.25)	4.36–4.78	0.7	3.6
SF-36v2					
	Physical function	78.51 (21.16)	74.95–82.08	0.7	23.2
	Role physical	75.96 (23.32)	72.03–79.88	0.7	36.2
	Bodily pain	69.78 (20.52)	66.32–73.23	2.2	19.6
	General health	57.42 (18.98)	54.23–60.62	0.7	0.7
	Vitality	55.75 (16.57)	52.97–58.54	1.4	0.7
	Social function	71.29 (22.73)	67.46–75.11	0.7	18.3
	Role emotion	75.42 (25.46)	71.18–79.71	0.7	41.3
	Mental health	63.37 (16.54)	60.59–66.15	0.7	1.4
	PCS	50.15 (6.84)	48.00–51.30	0.7	0.7
	MCS	44.83 (9.13)	43.30–46.37	0.7	0.7

PCS, physical component score; MCS, mental component score.

### Validity

Discriminant validity was assessed using known group comparisons. Every domain in Mal-PCOSQ was able to determine women who differed in PCOS-specific symptoms, hence supporting the validity of Mal-PCOSQ (**Table 4**). For convergence validity testing, a good correlation was found between similar domains of the two instruments used, namely, Mal-PCOSQ and SF-36v2 (**Table 5**). In the Mal-PCOSQ, the emotional domain correlated well with role emotion (*r* = 0.469; *p* < 0.001), social function (*r* = 0.464; *p* < 0.01), mental health (*r* = 0.476; *p* < 0.01), and mental component summary (*r* = 0.545; *p* < 0.01) of SF-36v2. These correlations were stronger than the other domains, e.g., physical function (*r* = 0.252). In the Mal-PCOSQ, the menstrual problem domain correlated with the bodily pain domain in the SF-36v2 (*r* = 0.439; *p* < 0.01). In the Mal-PCOSQ, the infertility role domain correlated significantly with role emotion in the SF-36v2 (*r* = 0.423; *p* < 0.01), thereby indicating that infertility has a significant impact on the emotions of women with PCOS.

**Table 4:** Mal-PCOSQ domain with different clinical parameters.

Domains	Clinical parameters		*P*-value
	FG score <8	FG score >8	
Body hair Mean (SD)	5.8 (1.1)	5.1 (1.7)	0.003*
	Normal BMI	Abnormal BMI	
Weight Mean (SD)	5.23 (1.5)	3.45 (1.3)	0.000*
	No infertility	Presence of infertility	
Infertility Mean (SD)	4.1 (1.6)	3.2 (1.4)	0.006*
	Regular menses	Irregular menses	
Menstrual Mean (SD)	5.1 (1.2)	4.2 (1.2)	0.017*

FG, Ferriman–Gallway; BMI, body mass index.

*p < 0.05.

**Table 5:** Construct validity: correlations between Mal-PCOSQ and SF-36 questionnaires.

SF-36v2	Mal-PCOSQ domains (correlation, *p*-value)				
	Emotions	Body hair	Weight	Infertility	Menstrual problem
Physical function	0.252 (0.003)	0.275 (0.001)	0.119 (0.163)	0.148 (0.082)	0.283 (0.001)
Role physical	0.372 (0.000)	0.215 (0.011)	0.232 (0.006)	0.319 (0.000)	0.384 (0.000)
Bodily pain	0.231 (0.007)	0.265 (0.002)	0.162 (0.058)	0.145 (0.089)	0.439 (0.000)
General health	0.328 (0.000)	0.266 (0.002)	0.249 (0.003)	0.295 (0.000)	0.367 (0.000)
Vitality	0.410 (0.000)	0.308 (0.000)	0.151 (0.078)	0.328 (0.000)	0.388 (0.000)
Social function	0.464 (0.000)	0.251 (0.003)	0.287 (0.001)	0.352 (0.000)	0.445 (0.000)
Role emotion	0.469 (0.000)	0.210 (0.013)	0.297 (0.000)	0.423 (0.000)	0.390 (0.000)
Mental health	0.476 (0.000)	0.284 (0.001)	0.228 (0.007)	0.387 (0.000)	0.357 (0.000)
PCS	0.219 (0.10)	0.286 (0.001)	0.154 (0.072)	0.138 (0.107)	0.389 (0.000)
MCS	0.545 (0.000)	0.259 (0.002)	0.294 (0.000)	0.469 (0.000)	0.414 (0.000)

PCS, physical component score; MCS, mental component score.

### Prediction Factor for the Mal -PCOSQ Subscale

Age can predict the scores of the emotion subscale (=0.192, *p* = 0.048) and infertility subscale (=0.209, *p* = 0.027) of the MAL-PCOSQ, which means that, in our population, age is an important low score predictor factor. The duration of infertility is also a predictor of the scores of the infertility subscale of MAL-PCOSQ (=-0.313, *p* = 0.001). The longer the duration of infertility was, the lower the score of the infertility subscale was (**Table 6**).

**Table 6:** Domains of Mal-PCOSQ with predictors of low score.

	Domains of Mal-PCOSQ, mean (SD)									
Groups	Emotions		Body hair		Weight		Infertility		Menstrual problem	
	CI	*p*-value	CI	*p*-value	CI	*p*-value	CI	*p*-value	CI	*p*-value
Age	0.192 (0.001 to 0.106)	0.048	0.043 (-0.046 to 0.073)	0.657	0.077 (-0.037 to 0.087)	0.429	0.209 (0.008 to 0.124)	0.027	0.169 (-0.006 to 0.094)	0.082
Duration of Infertility	-0.089 (-0.127 to 0.46)	0.354	-0.175 (-0.187 to 0.008)	0.070	0.004 (-0.099 to 0.102)	0.969	-0.313 (-0.255 to -0.066)	0.001	-0.069 (-0.110 to 0.052)	0.476

Logistic regression analysis.

CI, confidence interval.

### Comparison of the HRQOL of Malaysian Women With PCOS With Those in Other Countries

**Figure 1:**
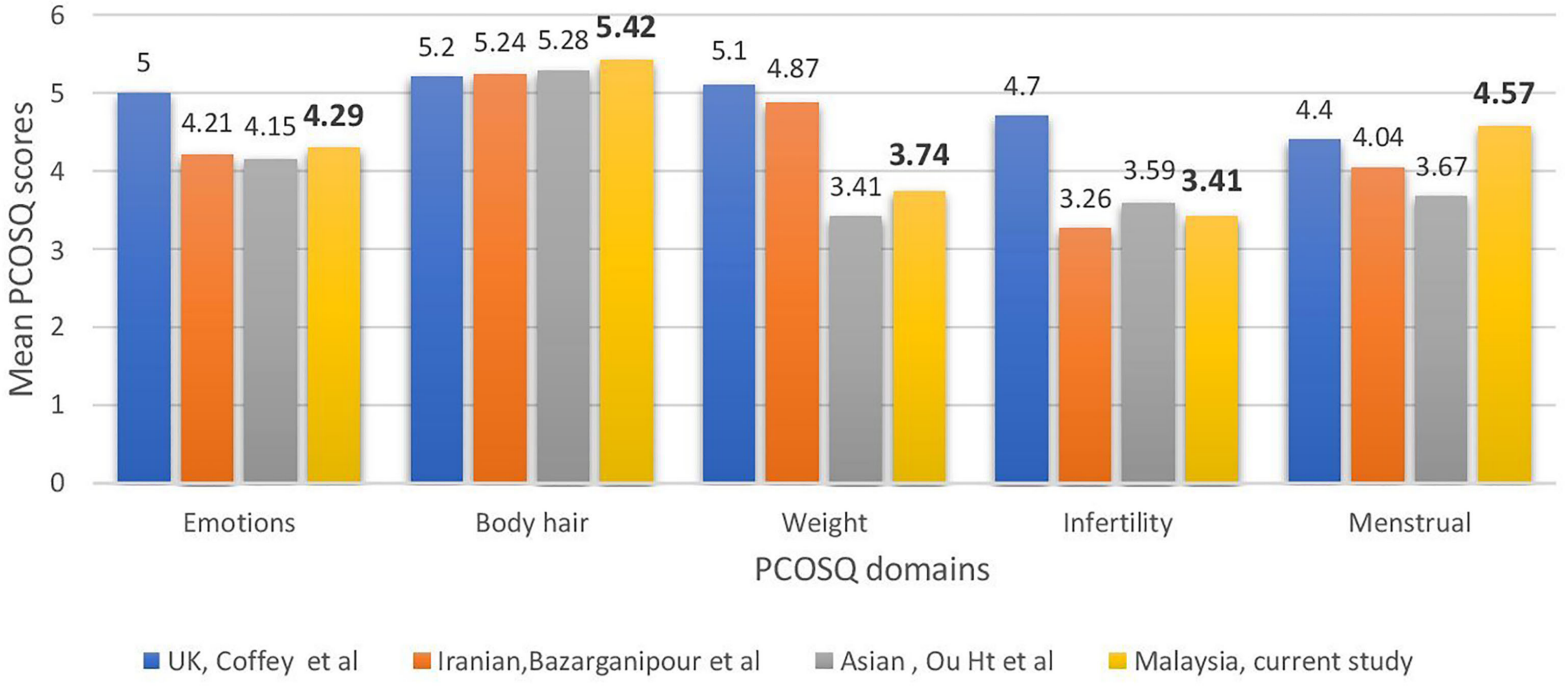
Comparisons across countries in health-related quality of life of polycystic ovary syndrome women measured using the Polycystic Ovarian Syndrome Questionnaire.

Compared with women of other ethnic origins, the affected domains varied significantly (**Figure 1**). In UK populations (22), weight and infertility appeared to be the main concerns, whereas the Iranian (20), Chinese (24), and Malaysian populations’ scores for infertility were the lowest. The Asian populations were the least concerned about body hair, unlike the UK population. This proved our hypothesis that PCOS affects various ethnicities differently.

## Discussion

The present study provided evidence of reliability and validity testing of Mal- PCOSQ. There was no dropout in our study, as it was applicable and relevant to the patients’ condition. None of the patients had difficulty understanding the questions in Malay, indicating that the Mal-PCOSQ could feasibly be used in the local population. All coefficients of the Mal-PCOSQ domains were analyzed using test– retest reliability analysis, which proved its good reliability with a Cronbach alpha score of >0.7 (average, 0.852). However, further modification regarding the menstrual problem domain is necessary as the alpha score was 0.678, which was in accordance with the previous studies (21, 22). Relocating certain non-specific questions, such as headache in the menstrual problem domain, was suggested to improve the questionnaire’s reliability.

The floor and ceiling effects were small in all subscales of the Mal-PCOSQ when compared with the SF-36v2. These findings indicated that the instrument was more specific towards certain areas pertaining to the disease or the condition itself, which was better than the generic instrument. The SF-36v2 questionnaire analysis also showed that both mental and physical score summaries were low, the former being 44.83 and the latter being 50.15. These results showed that our PCOS patients were significantly affected in terms of both mental and physical health, thereby supporting the findings of the Mal-PCOSQ and further enhancing its validity. Similar to the previous study by Coffey et al. (25), the convergent validity of the Mal-PCOSQ was proven by its correlation with similar domains in the SF-36v2 in which the “emotion” domain (Mal-PCOSQ) correlated well with the “role emotion” and “mental health” of the SF-36v2.

Known group comparisons, such as obesity, hirsutism, irregular menses, and subfertility, indicated that the Mal-PCOSQ was able to differentiate between subgroups with different clinical features, proving the discriminant validity of the instrument. A lower Mal-PCOSQ score was observed in women with PCOS with a higher Ferriman–Gallway score and body mass index and in those showing subfertility and irregular menses complications, in accordance with a previous study done by Chung et al. (23).

The present study also shows that Malaysian women with PCOS are affected by their condition, as evidenced by low scores in the infertility and weight domains. The local population was the least concerned about body hair. This was in contrast with the results of Cronin et al. (19), who found that the infertility and emotion subscales in the original PCOSQ had the highest impact scores among African-American women with PCOS. Huang et al. (24) showed that the burden of hirsutism on HRQoL among South Asians was less than that among Caucasians, with weight and infertility as the worst domains on the condition-specific questionnaire for both ethnic groups, according to the validated Chinese version of PCOSQ. The Iranian study (20) findings showed that Iranian patients were more affected by infertility and menstruation. The results of the present study were more similar to the study of Huang et al. (24), which included a similar Asian population. This finding proved the hypothesis that the impact of PCOS on quality of life varied among people of different ethnicities and backgrounds.

The Mal-PCOSQ can potentially be used in the diagnosis of the disease and during follow-ups to ascertain which component affects the patient the most and to assess the efficacy of the treatment provided. This will further improve patient care and outcome because each patient’s management will be tailored to their needs.

## Conclusion

The Mal-PCOSQ is a reliable and valid instrument that can be potentially used to assess the effect of PCOS on the quality of life and to assess the efficacy of treatment in follow-up consultations.

## Limitation of the Study

A limitation of this study is that it may not be representative of the Malaysian population as the participants were only recruited from one center. A further study can be done to include other centers to further investigate the strength of this questionnaire as well as to study the effect of PCOS on different populations, e.g., the indigenous group or adolescent group. A future study can also expand the domains of the Mal-PCOSQ by adding the acne subscale, which was proven to significantly affect the Chinese population.

## Data Availability Statement

The original contributions presented in the study are included in the article/supplementary material, further inquiries can be directed to the corresponding author.

## Ethics Statement

The studies involving human participants were reviewed and approved by the Research and Ethical Committee, Faculty of Medicine, University Kebangsaan Malaysia. The patients/participants provided their written informed consent to participate in this study.

## Author Contributions

LLM, MAA, and CKT designed the study and performed data collection. AI and AAZ analyzed the data. AGNA wrote the paper. All authors contributed to the article and approved the submitted version.

## Conflict of Interest

The authors declare that the research was conducted in the absence of any commercial or financial relationships that could be construed as a potential conflict of interest.

## Publisher’s Note

All claims expressed in this article are solely those of the authors and do not necessarily represent those of their affiliated organizations, or those of the publisher, the editors and the reviewers. Any product that may be evaluated in this article, or claim that may be made by its manufacturer, is not guaranteed or endorsed by the publisher.
