# Supplementary material for: The Reliability and Validity of the Malay Version of Polycystic Ovarian Syndrome Health-Related Quality of Life Questionnaire
Source: Front Endocrinol (Lausanne). 2022 May 26;13:848860. doi: 10.3389/fendo.2022.848860 (PMC9178788; doi:10.3389/fendo.2022.848860)
Supplement: Supplementary file 1 [file DataSheet_1.doc]

**APPENDIX 1 : SF 36 v2 MALAY VERSION HEALTH SURVEY**

# Kesihatan dan Kesejahteraan Anda

**Tinjauan ini meminta pandangan anda mengenai kesihatan anda. Maklumat ini akan memantau keadaan anda dan betapa baiknya anda dapat melakukan aktiviti anda yang biasa. *Terima kasih kerana melengkapkan tinjauan ini!***

**Untuk setiap soalan berikut, sila tandakan**  **di dalam satu kotak yang paling baik menerangkan jawapan anda.**

1. Secara umum, adakah anda akan mengatakan bahawa kesihatan anda adalah:

| Paling baik | Sungguh baik | Baik | Sederhana | Tidak baik |
| --- | --- | --- | --- | --- |
|  |  |  |  |  |
| 1 | 2 | 3 | 4 | 5 |

**2. Berbanding dengan setahun yang lalu, bagaimanakah anda menilai kesihatan anda secara umum sekarang?**

| Jauh lebih baik sekarang daripada setahun yang lalu | Agak lebih baik sekarang daripada setahun yang lalu | Lebih kurang sama dengan setahun yang lalu | Agak lebih teruk sekarang daripada setahun yang lalu | Jauh lebih teruk sekarang daripada setahun yang lalu |
| --- | --- | --- | --- | --- |
|  |  |  |  |  |
| 1 | 2 | 3 | 4 | 5 |

|  | Ya,  terbatas dengan banyaknya | Ya,  terbatas dengan sedikitnya | Tidak,  tidak  terbatas  sama sekali |
| --- | --- | --- | --- |
|  |  |  |  |
| a Aktiviti yang bertenaga dan sihat, seperti berlari, mengangkat barang berat, menyertai sukan yang  memerlukan tenaga dan kekuatan  1  2  3 | | | |
| b Aktiviti sederhana, seperti mengalihkan meja,  menyapu, bermain badminton atau bercucuk tanam  1  2  3 | | | |
| c Mengangkat atau membawa barang-barang runcit  1  2  3 | | | |
| d Menaiki beberapa larian tangga  1  2  3 | | | |
| e Menaiki satu larian tangga  1  2  3 | | | |
| f Membengkok, melutut atau membongkok  1  2  3 | | | |
| g Berjalan lebih daripada satu kilometer  1  2  3 | | | |
| h Berjalan beberapa ratus meter  1  2  3 | | | |
| i Berjalan seratus meter  1  2  3 | | | |
| j Mandi atau memakai pakaian sendiri  1  2  3 | | | |

**3.** **Soalan-soalan berikut adalah mengenai aktiviti yang mungkin akan anda lakukan pada hari biasa. Adakah anda terbatas dalam sebarang aktiviti berikut kerana keadaan kesihatan anda sekarang?
Jika ya, sejauh mana?**

4. Dalam masa 4 minggu yang lalu, berapa kerapkah anda mengalami sebarang masalah berikut apabila anda bekerja atau melakukan aktiviti harian tetap anda yang lain akibat kesihatan fizikal anda?

|  | Setiap  masa | Kebanyakan masa | Kadangkala | Sedikit  masa | Tiada sama sekali |
| --- | --- | --- | --- | --- | --- |
|  |  |  |  |  |  |
| a Mengurangkan jumlah masa  yang anda habiskan untuk  bekerja atau melakukan  aktiviti lain  1  2  3  4  5 | | | | | |
| b Mencapai kurang daripada  yang anda ingini  1  2  3  4  5 | | | | | |
| c Terbatas dari segi jenis  pekerjaan atau aktiviti lain  1  2  3  4  5 | | | | | |
| d Mempunyai kesukaran bekerja  atau melakukan aktiviti lain  (misalnya, memerlukan usaha  yang lebih)  1  2  3  4  5 | | | | | |

5. Dalam masa 4 minggu yang lalu, berapa kerapkah anda mengalami sebarang masalah berikut apabila anda bekerja atau melakukan aktiviti harian tetap anda yang lain akibat sebarang masalah emosi (seperti berasa murung atau bimbang)?

|  | Setiap  masa | Kebanyakan masa | Kadangkala | Sedikit  masa | Tiada sama sekali |
| --- | --- | --- | --- | --- | --- |
|  |  |  |  |  |  |
| a Mengurangkan jumlah masa  yang anda habiskan untuk bekerja  atau melakukan aktiviti lain  1  2  3  4  5 | | | | | |
| b Mencapai kurang daripada  yang anda ingini  1  2  3  4  5 | | | | | |
| c Bekerja atau melakukan aktiviti  lain dengan kurang berhati-hati  daripada biasa  1  2  3  4  5 | | | | | |

6. Dalam masa 4 minggu yang lalu, sejauh manakah kesihatan fizikal atau masalah emosi mengganggu aktiviti sosial biasa anda bersama keluarga, sahabat handai, jiran tetangga atau kumpulan?

| Tidak sama sekali | Sedikit | Sederhana | Agak banyak | Amat sangat |
| --- | --- | --- | --- | --- |
|  |  |  |  |  |
| 1 | 2 | 3 | 4 | 5 |

7. Dalam masa 4 minggu yang lalu, berapa banyakkah kesakitan yang anda alami?

| Tiada | Sedikit sangat | Sedikit | Banyak | Agak Banyak | Sungguh Banyak |
| --- | --- | --- | --- | --- | --- |
|  |  |  |  |  |  |
| 1 | 2 | 3 | 4 | 5 | 6 |

8. Dalam masa 4 minggu yang lalu, sejauh manakah kesakitan telah mengganggu pekerjaan biasa anda (termasuk pekerjaan di luar rumah dan kerja rumah)?

| Tidak sama sekali | Sedikit | Sederhana | Agak banyak | Amat sangat |
| --- | --- | --- | --- | --- |
|  |  |  |  |  |
| 1 | 2 | 3 | 4 | 5 |
|  |  |  |  |  |

9. Soalan-soalan ini adalah mengenai perasaan dan keadaan anda dalam masa 4 minggu yang lalu. Untuk setiap soalan, sila berikan satu jawapan yang paling hampir dengan keadaan perasaan anda. Dalam masa 4 minggu yang lalu, berapa kerapkah…

|  | Setiap  masa | Kebanyakan masa | Kadangkala | Sedikit  masa | Tiada sama sekali |
| --- | --- | --- | --- | --- | --- |
|  |  |  |  |  |  |
| a Adakah anda berasa penuh  bersemangat?  1  2  3  4  5 | | | | | |
| b Pernahkah anda berasa  sungguh gementar?  1  2  3  4  5 | | | | | |
| c Pernahkah anda berasa sungguh  sedih hinggakan tiada apa pun  yang dapat menceriakan anda?  1  2  3  4  5 | | | | | |
| d Pernahkah anda berasa tenang  dan aman?  1  2  3  4  5 | | | | | |
| e Adakah anda sungguh bertenaga?  1  2  3  4  5 | | | | | |
| f Pernahkah anda berasa sedih  dan murung?  1  2  3  4  5 | | | | | |
| g Adakah anda berasa sangat letih?  1  2  3  4  5 | | | | | |
| h Pernahkah anda berasa gembira?  1  2  3  4  5 | | | | | |
| i Adakah anda berasa letih?  1  2  3  4  5 | | | | | |

10. Dalam masa 4 minggu yang lalu, berapa kerapkah kesihatan fizikal atau masalah emosi telah mengganggu aktiviti sosial anda (seperti melawat sahabat handai, sanak saudara, dll.)?

| Setiap  masa | Kebanyakan masa | Kadangkala | Sedikit  masa | Tiada sama sekali |
| --- | --- | --- | --- | --- |
|  |  |  |  |  |
| 1 | 2 | 3 | 4 | 5 |
|  |  |  |  |  |

11. Sejauh manakah setiap penyataan berikut BENAR atau TIDAK BENAR untuk anda?

|  | Sungguh benar | Kebanyakan­nya benar | Tidak  tahu | Kebanyakan­nya tidak  benar | Sungguh tidak benar |
| --- | --- | --- | --- | --- | --- |
|  |  |  |  |  |  |
| a Saya kelihatan lebih mudah  jatuh sakit daripada orang lain  1  2  3  4  5 | | | | | |
| b Saya sihat seperti orang lain  yang saya kenali  1  2  3  4  5 | | | | | |
| c Saya menjangkakan kesihatan  saya akan menjadi lebih teruk  1  2  3  4  5 | | | | | |
| d Kesihatan saya adalah  sungguh baik  1  2  3  4  5 | | | | | |

*Terima kasih kerana melengkapkan soalan-soalan ini*

**APPENDIX 2 : Mal-PCOSQ Questionnaire**

**Sejauh mana anda berasa pertumbuhan bulu yang ketara pada dagu anda memberikan masalah kepada anda dalam tempoh dua minggu lepas**:

|  | Teramat bermasalah | Amat  bermasalah | Sederhana bermasalah | Agak bermasalah | Sedikit bermasalah | Hampir tiada masalah | Tiada masalah |
| --- | --- | --- | --- | --- | --- | --- | --- |
| 1. Pertumbuhan bulu yang ketara pada dagu? |  |  |  |  |  |  |  |

**Dalam tempoh dua minggu lepas**, berapa kali anda berasa:

|  | Setiap masa | Sangat kerap | Agak kerap | Kadang-kadang | Jarang | Jarang sekali | Tiada |
| --- | --- | --- | --- | --- | --- | --- | --- |
| 2. Murung kerana menghidap PCOS? |  |  |  |  |  |  |  |
| 3. Risau kerana berat badan berlebihan? |  |  |  |  |  |  |  |
| 4. Cepat letih? |  |  |  |  |  |  |  |
| 5. Risau dengan masalah ketidaksuburan? |  |  |  |  |  |  |  |
| 6. Angin tidak baik kerana menghidap PCOS? |  |  |  |  |  |  |  |

**Berhubung haid terakhir anda, sejauh mana isu yang berikut memberikan masalah kepada anda:**

|  | Teramat bermasalah | Amat  bermasalah | Sederhana bermasalah | Agak bermasalah | Sedikit bermasalah | Hampir tiada masalah | Tiada masalah |
| --- | --- | --- | --- | --- | --- | --- | --- |
| 7. Sakit kepala? |  |  |  |  |  |  |  |
| 8. Kitaran haid tidak teratur? |  |  |  |  |  |  |  |

**Sejauh mana pertumbuhan bulu yang ketara pada bibir atas anda memberikan masalah kepada anda dalam tempoh dua minggu lepas**:

|  | Teramat bermasalah | Amat  bermasalah | Sederhana bermasalah | Agak bermasalah | Sedikit bermasalah | Hampir tiada masalah | Tiada masalah |
| --- | --- | --- | --- | --- | --- | --- | --- |
| 9. Pertumbuhan bulu yang ketara pada bibir atas? |  |  |  |  |  |  |  |

**Dalam tempoh dua minggu** lepas, berapa kali anda:

|  | Setiap masa | Sangat kerap | Agak kerap | Kadang-kadang | Jarang | Jarang sekali | Tiada |
| --- | --- | --- | --- | --- | --- | --- | --- |
| 10. Menghadapi kesukaran dengan berat badan anda? |  |  |  |  |  |  |  |
| 11. Berasa rendah diri kerana menghidap PCOS? |  |  |  |  |  |  |  |
| 12. Berasa kecewa ketika cuba menurunkan berat badan? |  |  |  |  |  |  |  |
| 13. Berasa takut tidak dapat mengandungkan anak? |  |  |  |  |  |  |  |
| 14. Berasa takut akan menghidap kanser? |  |  |  |  |  |  |  |

**Dalam tempoh dua minggu lepas**, sejauh mana isu yang berikut memberikan masalah kepada anda:

|  | Teramat bermasalah | | Amat  bermasalah | | Sederhana bermasalah | Agak bermasalah | | Sedikit bermasalah | | Hampir tiada masalah | | Tiada masalah |
| --- | --- | --- | --- | --- | --- | --- | --- | --- | --- | --- | --- | --- |
| 15. Pertumbuhan bulu yang ketara pada muka? | |  |  |  | |  |  | |  | |  | |
| 16. Berasa malu kerana bulu badan berlebihan? | |  |  |  | |  |  | |  | |  | |

**Dalam tempoh dua minggu lepas, berapa kali anda:**

|  | Setiap masa | Sangat kerap | Agak kerap | Kadang-kadang | Jarang | Jarang sekali | Tiada |
| --- | --- | --- | --- | --- | --- | --- | --- |
| 17. Berasa risau kerana menghidap PCOS? |  |  |  |  |  |  |  |
| 18. Berasa malu kerana menghidap PCOS? |  |  |  |  |  |  |  |

**Berhubung haid terakhir anda**, sejauh mana isu yang berikut memberikan masalah kepada anda:

|  | Teramat bermasalah | | Amat  bermasalah | | Sederhana bermasalah | Agak bermasalah | | Sedikit bermasalah | | Hampir tiada masalah | | Tiada masalah |
| --- | --- | --- | --- | --- | --- | --- | --- | --- | --- | --- | --- | --- |
| 19. Kembung perut? | |  |  |  | |  |  | |  | |  | |
| 20. Haid lambat? | |  |  |  | |  |  | |  | |  | |
| 21. Senggugut? | |  |  |  | |  |  | |  | |  | |

**Dalam tempoh dua minggu lepas,** berapa kali anda:

|  | Setiap masa | Sangat kerap | Agak kerap | Kadang-kadang | Jarang | Jarang sekali | Tiada |
| --- | --- | --- | --- | --- | --- | --- | --- |
| 22. Berasa diri tidak seksi kerana berat badan berlebihan? |  |  |  |  |  |  |  |
| 23. Berasa kurang kawalan terhadap keadaan PCOS? |  |  |  |  |  |  |  |
| 24. Mempunyai kesukaran untuk mengekalkan berat badan ideal anda? |  |  |  |  |  |  |  |
| 25. Berasa sedih kerana mempunyai masalah ketidaksuburan? |  |  |  |  |  |  |  |

**Sejauh mana pertumbuhan bulu badan yang ketara memberikan masalah kepada anda dalam tempoh dua minggu lepas**:

|  | Teramat bermasalah | Amat  bermasalah | Sederhana bermasalah | Agak bermasalah | Sedikit bermasalah | Hampir tiada masalah | Tiada masalah |
| --- | --- | --- | --- | --- | --- | --- | --- |
| 26. Pertumbuhan bulu badan yang ketara? |  |  |  |  |  |  |  |

Terima kasih kerana melengkapkan soal selidik ini.
