# Supplementary material for: The Reliability and Validity of the Malay Version of Polycystic Ovarian Syndrome Health-Related Quality of Life Questionnaire
Source: Front Endocrinol (Lausanne). 2022 May 26;13:848860. doi: 10.3389/fendo.2022.848860 (PMC9178788; doi:10.3389/fendo.2022.848860)
Supplement: Supplementary file 2 [file DataSheet_2.docx]

Appendix 1

SF-36 QUESTIONNAIRE

# Name: ID#:

**Ref. Dr: Age:**

**Date: Gender: M / F**

Please answer the 36 questions of the **Health Survey** completely, honestly, and without interruptions.

# GENERAL HEALTH:

**In general, would you say your health is:**


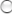
 Excellent
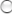
Very Good
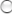
Good
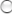
Fair
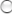
Poor

# Compared to one year ago, how would you rate your health in general now?


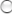

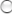

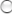

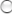

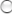


Much better now than one year ago Somewhat better now than one year ago About the same

Somewhat worse now than one year ago Much worse than one year ago

# LIMITATIONS OF ACTIVITIES:

The following items are about activities you might do during a typical day. Does your health now limit you in these activities? If so, how much?

# Vigorous activities, such as running, lifting heavy objects, participating in strenuous sports.


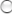
Yes, Limited a lot
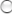
Yes, Limited a Little
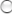
No, Not Limited at all

# Moderate activities, such as moving a table, pushing a vacuum cleaner, bowling, or playing golf


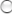
Yes, Limited a Lot
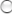
Yes, Limited a Little
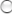
No, Not Limited at all

# Lifting or carrying groceries


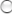
Yes, Limited a Lot
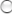
Yes, Limited a Little
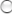
No, Not Limited at all

# Climbing several flights of stairs


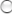
Yes, Limited a Lot
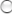
Yes, Limited a Little
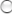
No, Not Limited at all

# Climbing one flight of stairs


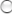
Yes, Limited a Lot
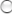
Yes, Limited a Little
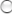
No, Not Limited at all

# Bending, kneeling, or stooping


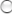
Yes, Limited a Lot
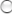
Yes, Limited a Little
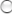
No, Not Limited at all

# Walking more than a mile


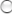
Yes, Limited a Lot
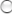
Yes, Limited a Little
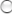
No, Not Limited at all

# Walking several blocks


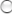
Yes, Limited a Lot
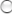
Yes, Limited a Little
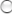
No, Not Limited at all

#
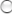

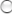

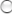
Walking one block

Yes, Limited a Lot Yes, Limited a Little No, Not Limited at all

# Bathing or dressing yourself


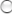
Yes, Limited a Lot
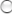
Yes, Limited a Little
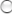
No, Not Limited at all

# PHYSICAL HEALTH PROBLEMS:

During the past 4 weeks, have you had any of the following problems with your work or other regular daily activities as a result of your physical health?

# Cut down the amount of time you spent on work or other activities


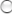
Yes
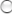
No

# Accomplished less than you would like


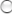
Yes
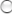
No

# Were limited in the kind of work or other activities


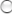
Yes
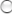
No

# Had difficulty performing the work or other activities (for example, it took extra effort)


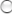
Yes
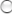
No

# EMOTIONAL HEALTH PROBLEMS:

During the past 4 weeks, have you had any of the following problems with your work or other regular daily activities as a result of any emotional problems (such as feeling depressed or anxious)?

# Cut down the amount of time you spent on work or other activities


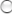
Yes
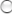
No

# Accomplished less than you would like


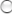
Yes
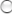
No

# Didn't do work or other activities as carefully as usual


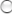
Yes
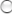
No

# SOCIAL ACTIVITIES:

**Emotional problems interfered with your normal social activities with family, friends, neighbors, or groups?**


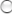
Not at all
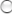
Slightly
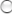
Moderately
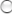
Severe
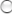
Very Severe

# PAIN:

**How much bodily pain have you had during the past 4 weeks?**


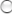
None
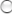
Very Mild
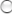
Mild
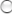
Moderate
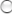
Severe
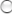
Very Severe

# During the past 4 weeks, how much did pain interfere with your normal work (including both work outside the home and housework)?


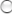

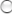

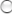

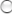

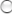
Not at all A little bit Moderately Quite a bit Extremely

# ENERGY AND EMOTIONS:

These questions are about how you feel and how things have been with you during the last 4 weeks. For each question, please give the answer that comes closest to the way you have been feeling.

# Did you feel full of pep?


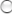

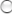

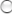

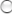

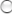

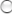


All of the time Most of the time

A good Bit of the Time Some of the time

A little bit of the time None of the Time

# Have you been a very nervous person?


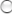

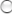

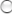

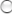

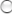

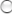


All of the time Most of the time

A good Bit of the Time Some of the time

A little bit of the time None of the Time

# Have you felt so down in the dumps that nothing could cheer you up?


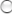

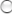

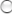

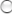

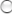

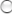


All of the time Most of the time

A good Bit of the Time Some of the time

A little bit of the time None of the Time

# Have you felt calm and peaceful?


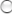

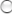

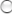

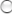

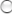

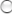


All of the time Most of the time

A good Bit of the Time Some of the time

A little bit of the time None of the Time

# Did you have a lot of energy?


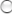

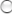

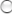

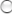

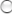

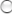


All of the time Most of the time

A good Bit of the Time Some of the time

A little bit of the time None of the Time

# Have you felt downhearted and blue?

All of the time Most of the time

A good Bit of the Time Some of the time

A little bit of the time None of the Time

# Did you feel worn out?

All of the time Most of the time

A good Bit of the Time Some of the time

A little bit of the time None of the Time

# Have you been a happy person?

All of the time Most of the time

A good Bit of the Time Some of the time

A little bit of the time None of the Time

# Did you feel tired?

All of the time Most of the time

A good Bit of the Time Some of the time

A little bit of the time None of the Time

# SOCIAL ACTIVITIES:

**During the past 4 weeks, how much of the time has your physical health or emotional problems interfered with your social activities (like visiting with friends, relatives, etc.)?**

All of the time Most of the time Some of the time

A little bit of the time None of the Time

# GENERAL HEALTH:

**How true or false is each of the following statements for you?**

**I seem to get sick a little easier than other people**

Definitely true Mostly true Don't know Mostly false Definitely false

# I am as healthy as anybody I know

Definitely true Mostly true Don't know Mostly false Definitely false

# I expect my health to get worse

Definitely true Mostly true Don't know Mostly false Definitely false

# My health is excellent

Definitely true Mostly true Don't know Mostly false Definitely false

**APPENDIX 2 : PCOSQ ORIGINAL VERSION**
